# Implementation of a New Discharge Card in the Department of Internal Medicine at Atbara Teaching Hospital

**DOI:** 10.7759/cureus.93250

**Published:** 2025-09-26

**Authors:** Mohammad Alzain Adam, Abdelmuniem Ahmed, Ayman Adil Eltayeb Abdelnour, Reem Saifeldin, Duaa Isameldin Abdelrazig Merghani, Abdelrahman Edris Osman Ali, Mojahed Abdallah Mohammed, Alshyma Alfatih Mohamed Abdallah, Ashraf Yousif Mohamed Alamin Yousif, Romisaa Elamin, Abdelwahab Ahmed, Noorallah Mohamed Babikir Abdalbagi, Mohamed Abdalla Elawad Wedatalla, Eman Esam Hassan Ali, Mariam Mohamed Musse Yusuf, Ahmed Magdi Hassan Elsayed, Tasneem Sulieman M Tahameed, Khobaib Abbas Abdin Osman, Imam Basheer, Abdelrahman Tageldin Osman Eltahir

**Affiliations:** 1 Internal Medicine, Sudan Medical Specialization Board, Khartoum, SDN; 2 Physiology, University of Gezira, Medani, SDN; 3 Internal Medicine, Kassala Teaching Hospital, Kassala, SDN; 4 Neonatology, Maternity and Children Hospital, Najran, SAU; 5 Pediatrics, Hamad Medical Corporation, Doha, QAT; 6 Internal Medicine, Atbara Teaching Hospital, Atbara, SDN; 7 Medicine, Sultan Qaboos University Hospital, Muscat, OMN; 8 General Surgery, Managil Teaching Hospital, Managil, SDN; 9 Accident and Emergency, AlJowhara Hospital, Al Ain, ARE; 10 General Surgery, Prince Othman Digna Teaching Hospital, Port Sudan, SDN; 11 Internal Medicine, Kassala University Hospital, Kassala, SDN; 12 General Medicine, Ministry of Health, Arar, SAU; 13 General Surgery, Atbara Teaching Hospital, Atbara, SDN; 14 Critical Care, Atbara Teaching Hospital, Atbara, SDN; 15 General Practice, University of Medical Sciences and Technology, Khartoum, SDN; 16 General Medicine, National Ribat University, Khartoum, SDN; 17 General Practice, Al-Neelain University Faculty of Medicine., Khartoum , SDN; 18 Medicine, Al-Manhal University Academy of Sciences, Khartoum North, SDN; 19 General Practice, Faculty of Medicine, Al-Neelain University, Khartoum, SDN

**Keywords:** discharge summary, documentation standards, internal medicine, patient safety, quality improvement project (qip)

## Abstract

Introduction: Effective discharge documentation is essential for safe transitions from hospital to community care. The Health Information and Quality Authority (HIQA) outlines seven core data categories for patient discharge summaries. In low-resource settings, structured formats have been shown to improve completeness, clarity, and patient safety. This quality improvement project (QIP) aimed to develop and implement a standardized discharge card in the Internal Medicine Department of Atbara Teaching Hospital, Sudan, adapted from HIQA standards.

Methodology: The project followed a two-cycle audit (April-August 2025). In the first cycle, 50 discharge records were reviewed to identify gaps. Records were randomly selected from the pool of available patient files, and auditing was conducted by three physicians not directly involved in patient care during the study period. A standardized discharge card was co-developed with staff, including essential demographic, clinical, and follow-up details. Training was provided, and the tool was implemented. In the second cycle, 40 discharge records were audited. Data was analyzed using chi-square tests to compare pre- and post-intervention documentation rates.

Results: Significant improvements were observed in gender documentation (46.8%→100%, *P *< 0.001), allergy recording (12.9%→88.3%, *P *< 0.001), telephone number (0%→36.6%, *P *< 0.001), address (1.6%→43.0%, *P *< 0.001), referral to specialist (0%→35.0%, *P *< 0.001), and referrer details (32.3%→55.0%, *P *= 0.028). High compliance was maintained for the patient’s name, age, primary diagnosis, and clinical summary. These gains are clinically meaningful, as improved allergy and contact documentation directly support patient safety and follow-up. Persistent deficiencies remained for date of birth (0%→5.0%, *P *= 0.110) and professional role documentation (40.3%→50.0%, *P *= 0.343), likely reflecting reliance on age notation and limited emphasis on role identification.

Conclusion: The standardized discharge card significantly improved documentation in key patient safety domains. Sustained gains require ongoing staff training and regular audits. This model may also be generalizable to other departments and hospitals in low-resource settings.

## Introduction

Effective discharge documentation is a critical component of patient care transitions from hospital to community or primary care settings. In 2013, the Health Information and Quality Authority (HIQA) in Ireland published a National Standard for Patient Discharge Summary Information to ensure safe, consistent, and comprehensive communication of clinical information at discharge [1]. This standard outline includes essential data grouped into seven categories: patient details, primary care healthcare professional details, admission and discharge information, clinical information, medication information, follow‑up and future management, and the details of the person completing the discharge summary. Implementing such structured discharge documentation facilitates continuity of care, reduces risks of errors, avoids unnecessary duplication of tests, and enhances patient safety after hospital discharge [1].

Several international studies reinforce that structured discharge documentation not only enhances inter-professional communication but also contributes to reductions in hospital readmissions and improved patient engagement. For example, a systematic review found that reorganizing discharge through better-quality information, coordinated care, and timely provider communication can reduce avoidable readmissions and associated costs [2]. Additionally, the implementation of the Patient-Oriented Discharge Summary (PODS), a co-designed, patient-centered discharge tool, was associated with improved teach-back, family involvement, and patient engagement in the discharge process [3].

Research in similar and low-resource settings further demonstrates the value of structured, standardized discharge processes. Continuous quality improvement projects (QIPs) to enhance paper-based discharge procedures have been shown to contribute significantly to better documentation and patient outcomes [4]. Clinical audits conducted in Sudanese hospitals reveal both persistent challenges and measurable improvements in discharge documentation completeness and accuracy of medication information [5-6]. Additional studies highlight that poor communication of discharge information can lead to adverse patient outcomes, missed follow-up, and medication errors [7-10]. Recent Sudanese experience with structured discharge summary formats illustrates that standardization significantly improves completeness and clarity of discharge information [11].

Therefore, this QIP aims to develop and implement a new discharge card in the Department of Internal Medicine at Atbara Teaching Hospital, guided by HIQA's national standards [1] and contextualized to local resources and needs. This initiative seeks to improve the quality, completeness, and clarity of discharge information, ensuring safer and more effective patient transitions from hospital to home or primary care follow-up. The central QIP question guiding this project was: Will the introduction of a structured, context-adapted discharge card improve the completeness and accuracy of discharge documentation compared to baseline practice?

## Materials and methods

Study design and setting

This QIP was done at Atbara Teaching Hospital, a tertiary health institution in Sudan that serves a heterogeneous population of both urban and rural residents. The project was focused on the internal medicine department, which had been identified as the area needing improvement in the discharge documentation process. The Internal Medicine Department has an approximate capacity of 60 beds and admits an average of 120-150 patients per month, reflecting a high and diverse patient load. The QIP was initiated on April 12, 2025, and is still ongoing, having completed the first stage of intervention and assessment as of August 1, 2025.

Study phases and interventions

First Cycle (Pre-intervention State and Root Cause Analysis) (April 12-June 12)

The analysis identified key deficiencies in the discharge documentation processes at Atbra Teaching Hospital, including the failure to have a standardized discharge card and an endemic failure to record critical patient information, including date of birth, contact information, and follow-up advice. For a more rounded understanding of these problems, a root cause analysis was undertaken, including extensive data gathering and analysis of current discharge records. For this purpose, fifty discharge records were reviewed consecutively to reflect routine practice. Data extraction was conducted by two independent reviewers, and inter-observer reliability was ensured through cross-checking of findings, with any discrepancies resolved by consensus. Consultations were undertaken with physicians, nurses, and administrative staff working at the hospital to capture their views and confirm identified root causes of problems. The results of these consultations were designed to inform improved understanding of difficulties related to the discharge process and inform the development of relevant solutions for future use.

Interventions (June 13-July 13)

A standardized discharge card was developed in collaboration with medical staff and hospital management, and the template is provided in the appendices (Appendix). This card included comprehensive fields spanning patient demographics (full name, gender, date of birth, address, telephone), clinical data (diagnosis, allergies, treatment, follow-up plan), and professional credentials (name, role, signature). To support implementation, three interactive training sessions were conducted over four weeks, each lasting approximately two hours. A total of 20 healthcare professionals, including physicians, nurses, and administrative staff, were trained. The sessions combined short lectures, case-based discussions, and practical demonstrations of using the discharge card. Training emphasized the importance of accurate documentation for patient safety and continuity of care. Implementation was monitored to evaluate early adoption and documentation quality.

*Second Cycle (Post-intervention Evaluation) (July 14*​​​​​*-August 1)*

 The evaluation tested the introduction of a standardized discharge card system at Atbara Teaching Hospital, and it found significant improvement in the quality of documentation, along with continued non-adherence in critical areas. To determine the maximum efficacy of the intervention, a detailed assessment was conducted on 40 randomly selected discharge cards using newly developed criteria that were standardized through iterative consensus meetings with two senior physicians and a nursing supervisor, and validated by piloting on ten discharge records before the main evaluation. These criteria took into account the views of trained healthcare providers, such as doctors, nurses, and clinical experts, to identify hindrances to regular documentation practice. The results found an appreciable improvement in performance regarding gender documentation (100%) and allergy documentation (88.3%), and in addition, highlighted continued problems with date of birth documentation (5% adherence), address documentation (43%), and professional role identification (50%), thus providing operational recommendations for future improvement.

Data analysis

Discharge cards were randomly selected and evaluated by three physicians trained in standardized assessment protocols to ensure consistency. Documentation completeness and adherence to updated standards were analyzed using quantitative and qualitative methods, supplemented by stakeholder feedback. While randomization and trained reviewers strengthened reliability, the absence of documented blinding during evaluations introduced potential bias, a limitation affecting interpretation. The findings provided clinically valid insights for targeted improvements; however, future studies should incorporate blinding to further reduce bias.

Evaluation

Feedback and Effectiveness Assessment

The impact of the intervention was assessed by comparing documentation completeness and compliance rates before and after the introduction of the standardized discharge card. Structured feedback from healthcare providers and patients was used to identify ongoing challenges and inform future improvements. Patient feedback was systematically collected through short exit interviews with 30 recently discharged patients, focusing on clarity of discharge instructions, understanding of medication regimens, and awareness of follow-up plans. Responses were thematically analyzed by two independent reviewers to identify recurring gaps and strengths in communication.

Ethical Considerations

Ethical and managerial approval for the QIP was obtained from Atbara Teaching Hospital (approval number 2025-QI-008). All personal and clinical data were anonymized to protect patient privacy and confidentiality. As this was a QIP that involved analysis of existing documentation and staff training without direct patient intervention, individual informed consent was not required in accordance with institutional policy and international QI/QA ethical guidelines.

## Results

A total of 50 patient discharge records were reviewed in the first audit cycle and 40 in the second cycle. The comprehensiveness of documentation was assessed across multiple parameters to evaluate the impact of interventions conducted between cycles. Baseline patient demographics, including age distribution, gender ratio, and admission types, were broadly similar between the two cycles, minimizing the likelihood that demographic differences would confound the observed changes in documentation rates.

Patient’s Name remained highly documented in both cycles, with compliance dropping slightly from 98.4% to 97.0%. This minor change was not statistically significant (*P* = 0.873), indicating sustained performance and adherence to this basic standard. Date of birth was recorded in none of the first cycle records and only 5.0% of the second cycle. Despite a small improvement (+5.0%), this parameter remains under-documented, and the change was not statistically significant (*P* = 0.110). Patient’s age documentation stayed above 95% in both cycles with no significant difference (*P* = 0.819), reflecting consistent reliability for this essential demographic field.

Gender documentation rose dramatically from 46.8% to 100% (*P* < 0.001), indicating a targeted and highly effective intervention. Telephone Number also saw significant improvement, increasing from no documentation to 36.6% (*P* < 0.001). This enhancement is pivotal for post-discharge patient contact and continuity of care. Address documentation showed substantial improvement as well, from a negligible 1.6% to 43.0% (*P* < 0.001), strengthening the ability to follow up with patients and facilitate referrals.

Reason for Admission saw a modest decline from 95.2% to 90.0% (*P* = 0.257). This suggests possible lapses in standard recordkeeping, but the change was not statistically significant. Primary diagnosis was consistently documented in 100% of both cycles, marking it as a strong area of compliance requiring no additional intervention. The completeness of the clinical summary improved slightly from 93.5% to 96.6%, a gain of 3.1% that was not statistically significant (*P* = 0.423).

Allergies documentation underwent a major transformation, climbing from 12.9% to 88.3% (*P* < 0.001). This finding represents a critical enhancement for patient safety by reducing the risk of adverse reactions. Investigation results improved from 85.0% to 90.0%, but this 5% increase was not statistically significant (*P* = 0.405). Discharge Medications/Devices documentation dropped from 96.8% to 90.0% (*P* = 0.257). Although this decline was not statistically significant, it is essential to ensure consistent medication documentation for safe patient transitions. The follow-up plan improved modestly from 88.7% to 93.3% (+4.6%), with no statistical significance (*P* = 0.480).

Referral to a Specialist rose from zero documentation to 35.0% (*P* < 0.001), a large and statistically significant improvement that is vital for continuity of post-discharge care. Referrer Details increased from 32.3% to 55.0% (*P* = 0.028), also showing a significant improvement that supports seamless care coordination. Professional Role/Designation saw a 9.7% increase from 40.3% to 50.0%, though this was not statistically significant (*P* = 0.343).

Overall, parameters related to patient contact information, allergy status, and referral processes demonstrated the most substantial and statistically significant improvements, highlighting the effectiveness of targeted interventions in these areas (Table 1). High-performing fields such as Patient’s Name, Age, Primary Diagnosis, and Clinical Summary remained stable, reflecting ongoing compliance. However, persistently low documentation rates for some parameters, including Date of Birth and specific care details, indicate that further education, process optimization, or electronic system enhancements may be necessary to achieve comprehensive and consistent record-keeping across all domains.

**Table 1:** Documentation compliance before and after implementation of a standardized discharge card in the Department of Internal Medicine at Atbara Teaching Hospital. This table compares documentation parameters across two audit cycles: the first cycle (pre-intervention) and the second cycle (post-intervention) following the implementation of a standardized discharge card. The data represent the number and percentage of records with completed documentation. Improvement indicates the percentage point change between cycles. *P*-values were calculated using Chi-square tests to assess statistical significance (*P* < 0.05 considered significant).

Parameter	First cycle	Second cycle	Improvement	Chi-square (χ²)	*P*-value	Note
Patient’s Name	49 (98.4%)	39 (97.0%)	-1.4%	0.026	0.873	No significant change
Date of Birth	0 (0.0%)	2 (5.0%)	+5.0%	2.57	0.110	No significant change
Patient’s Age	48 (95.2%)	38 (95.0%)	-0.2%	0.05	0.819	No significant change
Gender	23 (46.8%)	40 (100.0%)	+53.2%	24.8	<0.001	Significant improvement
Telephone Number	0 (0.0%)	15 (36.6%)	+36.6%	18.3	<0.001	Significant improvement
Address	1 (1.6%)	17 (43.0%)	+41.4%	20.5	<0.001	Significant improvement
Reason for Admission	48 (95.2%)	36 (90.0%)	-5.2%	1.28	0.257	No significant change
Primary Diagnosis	50 (100.0%)	40 (100.0%)	+0.0%	–	–	No significant change
Clinical Summary	47 (93.5%)	39 (96.6%)	+3.1%	0.64	0.423	No significant change
Allergies	6 (12.9%)	35 (88.3%)	+75.4%	32.1	<0.001	Significant improvement
Investigation Results	42 (85.0%)	36 (90.0%)	+5.0%	0.70	0.405	No significant change
Discharge Medications/Devices	48 (96.8%)	36 (90.0%)	-6.8%	1.28	0.257	No significant change
Follow-up Plan	44 (88.7%)	37 (93.3%)	+4.6%	0.50	0.480	No significant change
Referral to a Specialist	0 (0.0%)	14 (35.0%)	+35.0%	16.5	<0.001	Significant improvement
Referrer Details	16 (32.3%)	22 (55.0%)	+22.7%	4.84	0.028	Significant improvement
Professional Role/Designation	20 (40.3%)	20 (50.0%)	+9.7%	0.89	0.343	No significant change

## Discussion

The goal of this QIP was to improve the quality, thoroughness, and readability of discharge paperwork by using a standard discharge card in the Department of Internal Medicine at Atbara Teaching Hospital. Hospital discharge represents a particularly vulnerable transition in patient care, where incomplete or inaccurate information can result in delays, omission of essential treatments, or unnecessary duplication of care. Previous studies have shown that nearly one in five patients experience adverse events following discharge, with almost one-third of these considered preventable [12,13]. The two rounds of audits showed marked improvements in several important areas, particularly in ensuring continuity of care and protecting patient safety. There were substantial increases in gender documentation, from 46.8% to 100% (*P* < 0.001), and in allergy documentation, which rose from 12.9% to 88.3% (*P* < 0.001). Gender documentation is particularly critical in Sudan’s healthcare context, as it informs gender-specific clinical decision-making, supports accurate reporting for health statistics, and ensures culturally appropriate continuity of care. Collectively, these improvements are likely to contribute to a reduction in preventable adverse post-discharge outcomes.

There was a big improvement in contact information, as shown by the fact that the number of people who filled out their phone numbers went from 0% to 36.6% (*P* < 0.001) and the number of people who filled out their addresses went from 1.6% to 43.0% (*P* < 0.001). Additionally, documentation of referral to special services improved from 0% to 35.0% (*P* < 0.001), and documentation of referrer information improved from 32.3% to 55.0% (*P* = 0.028). These improvements in referral and referrer documentation have important implications for continuity of care. By ensuring clear pathways to specialist services and confirming the responsible provider, such enhancements can reduce avoidable hospital readmissions, strengthen chronic disease follow-up, and promote more seamless coordination between hospital and community care.

The findings recorded are in accord with the National Standard for Patient Discharge Summary Information set by HIQA, which stipulates that the incorporation of thorough patient identifiers, relevant clinical information, and contact information is crucial in ensuring safe and effective continuity of care [1]. During each cycle, the details of patient name, age, main diagnosis, and clinical summary were carefully recorded since earlier research established that basic clinical information attains a high degree of consistency before the creation of any formal standardization system [7,14]. The findings recorded are in accord with the National Standard for Patient Discharge Summary Information set by HIQA, which stipulates that the incorporation of thorough patient identifiers, relevant clinical information, and contact information is crucial in ensuring safe and effective continuity of care [1]. During each cycle, the details of patient name, age, main diagnosis, and clinical summary were carefully recorded since earlier research established that basic clinical information attains a high degree of consistency before the creation of any formal standardization system [7,14]. This pattern reflects the fact that certain fields-such as patient name and age-are integral to routine admission processes and are usually captured at registration, making them consistently available. In contrast, details like date of birth and contact information often depend on either patient recall or staff emphasis at discharge, which may explain their persistent under-documentation and highlight the need for targeted reinforcement through training and system redesign.

The heightened emphasis on the optimization of the documentation of allergies is similarly important because poor allergy records have been linked to medication errors and adverse reactions to medications in all healthcare settings, irrespective of resource constraints [9,10]. The decline may be partly attributable to systemic challenges, including staff fatigue and time pressures during busy admission and discharge periods. Furthermore, training sessions emphasized demographic and allergy documentation as priority areas, which may have inadvertently diverted attention from ensuring consistent recording of admission reasons and discharge medications. Addressing these gaps in future cycles will require balanced training content and strategies to reduce staff workload at critical points of documentation. Similarly, increased documentation of contact details has been shown to enable continuity of care and follow-up after discharge [15]. These improvements are likely due to the personalized prompts included in the new discharge card, as well as the expert training materials provided to clinical staff, consistent with positive outcomes in other structured documentation programs in Sudan and similar settings [4,8,11]. Although this project did not systematically measure patient-level outcomes, anecdotal reports from staff suggested fewer prescribing uncertainties related to allergy status and improved success in arranging follow-up calls, highlighting the potential for these process improvements to translate into safer and more effective post-discharge care.

Despite these achievements, various fields of documentation showed little improvement. Documentation of date of birth only showed a negligible improvement from 0% to 5% (*P* = 0.110), and this area may have been missed or de-prioritized by staff, possibly because of poor data capture in the previous admissions pro forma. Professional fulfillment of an appointment or job improved marginally from 40.3% to 50% (*P* = 0.343), and more efforts are needed to ensure this, as it has important implications for responsibilities and proper information disclosure with providers on discharge. Unexpectedly, documentation of discharge medications and devices worsened from 96.8% to 90% (*P* = 0.257). This pattern is of concern because missing medication records are known to be an important risk factor for adverse outcomes after discharge [9-10]. Although this decline was not statistically significant, it remains clinically important, as incomplete or missing medication records pose a direct risk to patient safety by increasing the likelihood of dosing errors, omissions, or harmful drug interactions. This underscores the need for continuous reinforcement of medication documentation practices as a non-negotiable safety priority in future cycles.

Feedback from staff suggested that this decline may have been influenced by documentation fatigue during busy discharge periods, as well as limited emphasis on medication fields during the training program. In addition, systemic workflow barriers-such as reliance on verbal communication of prescriptions and time constraints in the pharmacy-ward interface-may have contributed to incomplete documentation. Addressing these issues in future cycles will require balanced training content and workflow redesign to prioritize medication documentation as a critical patient safety domain. Moreover, Claassen et al. reported that utilizing telephone-based follow-up procedures can maximize patient data retention in longitudinal studies [16], suggesting that similar strategies could be adopted to enhance documentation completeness in future cycles.

These findings confirm that even though paper-based designs were deliberately crafted, they significantly improve documentation of discharge across key safety fields; however, continued and comprehensive compliance requires continued investment in nursing staff, regular auditing and feedback processes, and integration into established processes. Analogous challenges to achieving universal compliance have been reported across limited resources Sudanese hospitals and wealthy health care settings [4,8].

There are several constraints identified in the research. The sample size was small, consisting of 50 records in the first cycle and 40 in the second, thereby limiting statistical power for some of the metrics. Reviewers were not blinded to the intervention phase and thus were at risk of potential bias. Follow-up was of short duration, limiting the assessment of the sustainability of changes identified in the long term. Evaluating whether these improvements can be maintained over several months or years is essential to demonstrate their durability and ensure long-term integration into routine clinical practice. Furthermore, informed consent documentation was lacking among patients, and this requires improvement in future QIPs. Future research should also assess whether these improvements in documentation lead to measurable patient outcomes, such as reduced readmission rates, fewer medication errors, and improved patient satisfaction.

## Conclusions

Implementation of a structured discharge card at Atbara Teaching Hospital’s Internal Medicine Department led to substantial and statistically significant improvements in several critical areas, including allergy status, contact information, and referral documentation. These enhancements align with international discharge documentation standards and are likely to improve continuity of care and patient safety. However, persistent low compliance in certain fields, particularly date of birth and professional role, indicates the need for targeted education and process reinforcement. Sustaining these improvements will require ongoing auditing, feedback, and potentially transitioning to an electronic discharge system to ensure completeness and accuracy across all parameters. Importantly, this intervention has the potential to be scaled to other departments and hospitals in Sudan and comparable low-resource contexts. Its broader adoption may be facilitated by the low cost and simplicity of paper-based tools, though barriers such as staff workload, training demands, and variable institutional support must be addressed to ensure successful implementation.

## Figures and Tables

**Figure 1:**
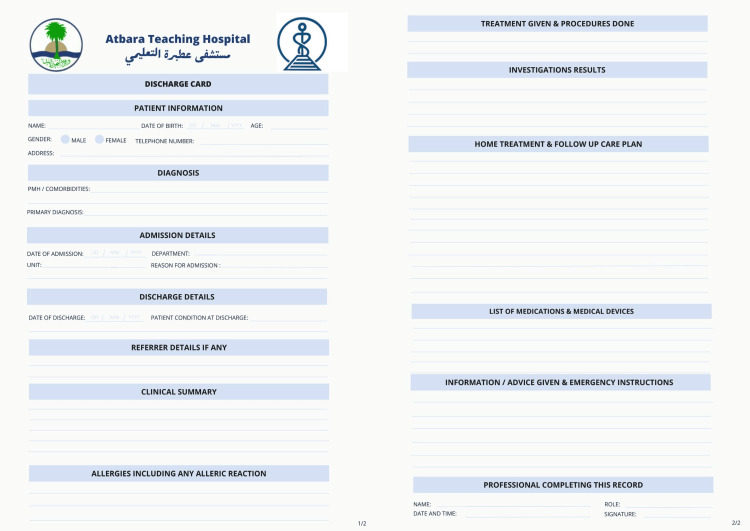
Standardized Discharge Card Template Used at Atbara Teaching Hospital This figure presents the official discharge card template adopted by Atbara Teaching Hospital. The card includes sections for patient information, diagnosis, admission and discharge details, treatment provided, investigation results, home treatment and follow-up plan, medication list, allergies, and professional documentation. It is designed to ensure comprehensive communication of patient care at the time of discharge.

## Appendices

Appendix 
